# Selective Increase of Auditory Cortico-Striatal Coherence during Auditory-Cued Go/NoGo Discrimination Learning

**DOI:** 10.3389/fnbeh.2015.00368

**Published:** 2016-01-11

**Authors:** Andreas L. Schulz, Marie L. Woldeit, Ana I. Gonçalves, Katja Saldeitis, Frank W. Ohl

**Affiliations:** ^1^Department Systems Physiology, Leibniz Institute for NeurobiologyMagdeburg, Germany; ^2^Department Systems Biology, Institute of Biology, Otto-von-Guericke UniversityMagdeburg, Germany; ^3^Center for Behavioral Brain SciencesMagdeburg, Germany

**Keywords:** auditory cortex, ventral striatum, discrimination learning, avoidance learning, field-field coherence, functional coupling, shuttlebox, Mongolian gerbil

## Abstract

Goal directed behavior and associated learning processes are tightly linked to neuronal activity in the ventral striatum. Mechanisms that integrate task relevant sensory information into striatal processing during decision making and learning are implicitly assumed in current reinforcement models, yet they are still weakly understood. To identify the functional activation of cortico-striatal subpopulations of connections during auditory discrimination learning, we trained Mongolian gerbils in a two-way active avoidance task in a shuttlebox to discriminate between falling and rising frequency modulated tones with identical spectral properties. We assessed functional coupling by analyzing the field-field coherence between the auditory cortex and the ventral striatum of animals performing the task. During the course of training, we observed a selective increase of functional coupling during Go-stimulus presentations. These results suggest that the auditory cortex functionally interacts with the ventral striatum during auditory learning and that the strengthening of these functional connections is selectively goal-directed.

## 1. Introduction

Learning of goal-directed behaviors relies on the evaluation of sensory stimuli. While many studies have demonstrated learning-induced modification of sensory processing in sensory cortical areas (e.g., Ohl et al., 2001; Weinberger, 2004; Froemke et al., 2007) as well as in the striatum (e.g., Doya et al., 2002; O'Doherty et al., 2004; Balleine et al., 2007; DeCoteau et al., 2007), studies investigating the conversion of sensory information to modified behaviors are still scarce. All sensory modalities evoke responses in the striatum (Glynn and Ahmad, 2002): visual (Schulz et al., 2009, 2011), tactile (Pidoux et al., 2011; Syed et al., 2011), olfactory (Wieland et al., 2015), and auditory (LeDoux et al., 1991; Cromwell et al., 2007; Woldeit et al., 2012). Sensory cortico-striatal connections do not constitute a concise topographical mapping, instead such projections are characterized by a high degree of convergence and divergence, as well as co-integration with motor and prefrontal cortical input (McGeorge and Faull, 1989; Voorn et al., 2004) and multisensory integration (Hikosaka et al., 1989; Nagy et al., 2005, 2006; Cui et al., 2013; Reig and Silberberg, 2014). Cortico-striatal connections have been postulated as a crucial circuit for the transformation of sensory information into decisions for behavioral options (Houk and Wise, 1995; Schultz and Dickinson, 2000; Amemori et al., 2011; Znamenskiy and Zador, 2013). However, neither is there much known about the exact neural mechanisms, nor has the functional connectivity between sensory cortex and striatum been systematically investigated. One functional study did not see any changes of coupling between the auditory cortex and striatum in the gamma frequency range during Pavlovian learning, but unfortunately did not extend their investigation to other frequency ranges during this task (Popescu et al., 2009).

A major experimental problem for the investigation of plasticity is the identification of the subset of cortico-striatal connections relevant in a particular learning scenario. Investigating auditory discrimination learning, Xiong et al. (2015) solved this problem in an elegant study by exploiting the tonotopic organization of the auditory cortico-striatal projections in rodents. Using optogenetics, they selectively targeted high- or low-frequency populations of neurons in the auditory cortex and corresponding neurons in the striatum. This method allowed the identification of specific subsets of cortico-striatal connections, in spatially non-overlapping high- and low-frequency projections between auditory cortex and dorsal striatum, which were selectively potentiated during learning. The authors concluded that the selective strengthening of cortico-striatal synapses might reflect a general mechanism by which sensory representations guide the selection of motor responses. They further suggested that this mechanism might also be active if the sensory stimuli cannot be separated topographically by their cortico-striatal projections.

To test this hypothesis an experimental paradigm is required in which sensory stimuli evoke largely overlapping sensory cortical representations and must be associated with different behavioral actions. According to these requirements we have studied discrimination learning of rising and falling frequency- modulated tones traversing an identical frequency interval. We have previously shown that such stimuli produce overlapping representations in primary auditory cortex (Ohl et al., 2000) but can nevertheless be discriminated and categorized (Ohl et al., 2001). To ensure a high contrast between goal-directed behaviors actuated by the semantic analysis of these stimuli, animals were trained in a Go/NoGo discrimination paradigm. In order to identify the relevant subpopulations of neurons in the auditory cortex and the striatum and to track plasticity between them we measured the stimulus-evoked neural coherency between primary auditory cortex and ventral striatum while animals performed the task. To our knowledge this is one of the first studies that directly measures sensory cortico-striatal functional connectivity during learning.

Using this approach we demonstrate that auditory stimuli activating tonotopically overlapping cortical areas modulate cortico-striatal coupling differentially when these stimuli are associated with specific goal-directed behaviors in an auditory discrimination task.

## 2. Materials and methods

### 2.1. Animal subjects

All experiments were performed in seven male Mongolian gerbils (*Meriones unguiculatus*), 3–5 months of age. All procedures were performed in accordance with the European Communities Council Directive of November 24, 1986 (86/609/EEC), and according to the German guidelines for the care and use of animals in laboratory research. Experiments were approved by the Ethics Committee of the state Saxony-Anhalt.

### 2.2. Electrode implantations

Surgical procedures are described in Woldeit et al. (2012). Briefly, gerbils were anesthetized with Nembutal (initial 50 mg/kg intraperitoneally, Sigma-Aldrich, St. Louis, MO, USA) and mounted into a stereotaxic frame. A custom-made surface electrode array (4 × 4 stainless steel electrodes, 100 μm single contact diameter, impedance range: 0.2–0.6 MOhm) was placed on the dura above the right auditory cortex as described in Ohl et al. (2000). For the array placement the vascularization pattern of the inferior cerebral vein and the middle cerebral artery were used as topographic landmarks (cf. Ohl et al., 2000).

A depth electrode array (4 bundles of two twisted micro wires, stainless-steel, 50 μm diameter per single wire, impedance range: 0.4–0.7 MOhm) was stereotaxically lowered into the ventral striatum of the same hemisphere (anterio-posterior: +0.5 mm, medio-lateral: −1.3 mm, dorso-ventral: −4.1 mm from bregma). A stainless steel screw in the frontal bone served as reference electrode for both arrays. Dental resin and further anchoring screws were used to secure the wiring and fix electrical connectors (Molex, USA) to the skull. Following surgery, animals were allowed at least 5 days for recovery.

### 2.3. Discrimination learning task

Gerbils were trained in a Go/NoGo discrimination task in a shuttlebox (Wetzel et al., 2008; shuttlebox system 38 × 19 × 22.5 cm; Hasomed GmbH, Magdeburg, Germany), inside a sound-proof and electrically shielded chamber. Animals learned to shuttle from one compartment to the other as response to a CS+ (a sequence of rising FM tones, 1–2 kHz, duration 200 ms, onset-onset inter-tone interval 0.5 s) in a period of 6 s in order to avoid a mild electrical foot shock applied via a metal grid floor (300 μA). Animals learned to stay in the ipsilateral compartment as response to a CS− (trains of FM tones 2–1 kHz): Shuttling within a period of 10 s resulted in an electrical foot shock. A successful shuttling in response to CS+ was called a hit, fail of shuttling during this condition a miss. Shuttling in response to CS− was scored as false alarm and a successfully staying in the ipsilateral compartment a correct rejection. Animals were trained daily with a pseudo-randomized order of 30 CS+ and 30 CS− trials. Sessions started with a pre-session period of 60–80 s without presentation of acoustical signals. These pre-sessions were used to assess baseline functional coupling.

### 2.4. Electrophysiological recording

During each training session, electrocorticograms (ECoG) and local field potentials (LFP) were recorded with a MAP recording system (Plexon Inc., Dallax, TX, USA) to which the animals were connected via a movable tether. ECoG and field potentials were filtered between 0.7 and 300 Hz and digitized at 1 kHz. Auditory stimuli were generated in MATLAB (Mathworks, Natick, TX, USA) and presented inside the chamber with an audio amplifier and electrostatic speaker (SRM313 and modified SR-307, Stax Ltd., Japan; average free-field sound pressure level amounted to 75 dB). The frequency response of the speaker was flat +/− 3 dB between 0.5 and 2 kHz.

### 2.5. Histological analysis and retrograde tracing

After termination of experiments, iron deposits were produced at the tip of the striatal electrodes via delivery of constant-current pulses (stimulator: STG 1008, Multi Channels Systems, Reutlingen, Germany; four rectangular pulses, 5 μA for 25 s each) to the awake animals. Afterwards, animals were deeply anesthetized and sacrificed with an intracardial injection of 0.5 ml T61 (Intervet GmbH, Germany). To determine electrode locations, brains were cut with a cryostat into 40 μm histological slices and subjected to Nissl and Prussian blue iron staining. Electrode location of the striatal arrays were verified with a gerbil brain atlas (Loskota et al., 1974).

To demonstrate direct connections between the ventral striatum and the auditory cortex, two animals were anesthetized (10 mg ketamine/100 g body weight, Ratiopharm GmbH, and 0.5 mg xylazine/100 g body weight, Bayer, intraperitoneal) and injected bilaterally into the ventral striatum with fluorescent nanobeads (RedRetrobeads IX, Lumafluor, excitation max: 530 nm, emission max. 590 nm, 50 μl dissolved in 3 ml of 0.01 M phosphate-buffer (PBS, pH 7.4), injection volume: 27.6 nl) that were additionally conjugated with Chlorine-e6-monoethylene-diamine-monoamide-disodium-salt (Phytochlorin, Frontier Scientific). Animals were also used for a different histological experiment; for the purpose of tracing the anatomical connections between AC and striatum the left hemispheres were analyzed.

Injections were placed in the ventral striatum at anterio-posterior: +0.77 mm, medio-lateral: ± 1.5 mm and dorso-ventral: −4.2 mm below the dura, using a nanoinjector (WPI). Following the injections, the cranial opening was closed with bone wax (Ethicon), and the skin was sutured and closed with tissue adhesive (Histoacryl, Braun).

After 20 days animals were deeply anesthetized and perfused with phosphate buffered saline (PBS, 0.1 M) and 4% paraformaldehyde dissolved in PBS. After post-fixation in 4% paraformaldehyde and cryoprotection in a 30% sucrose-PBS solution, 50 μm thick horizontal slices were cut with a cryostat. Every third slice was coverslipped in Mowiol for analysis of the fluorescent beads. Every fourth slice was treated according to NeuN staining protocol, using a monoclonal mouse antibody to NeuN and appropriate secondary biotinylated antibodies (anti-host IgG 1:200, Vector Labs). The reaction product was visualized by incubating the sections in the Avidin-Biotin Complex (Vectastain Elite ABC Kit, Vector Labs) and using 3,3-diaminobenzidine (0.4 mM, Sigma-Aldrich) as chromogen in the presence of 0.015% H_2_O_2_. The sections were mounted on gelatin-coated slides, dehydrated and then coverslipped.

Photographs of the auditory cortex were made using a Leica DFC 500 camera mounted on a microscope (Zeiss Axioskop 2), fitted with the appropriate filters for fluorescence (Leica filter N2.1, excitation filter: BP 515–560 nm).

### 2.6. Field-field coherence analysis

Electrode channels and trials showing artifacts were rejected for the analysis. One animal was not included in the population analysis as its coherence values (mCoh > 0.9) indicated a significant inter-electrode crosstalk. For one animal the last session was discarded due to poor signal-to-noise levels. Every session contained 60 trials. On average, 53 sessions were included for the coherence analysis, the rest were excluded due to artifacts in the LFP signal (minimum of used trials: 35 trials/session, maximum: 60 trials/session, median: 55 trials/session).

The ECoG recorded from the surface array represents a spatio-temporal superposition of the signals generated by two main dipoles within the auditory cortex that also includes potential anatomical cortico-striatal projections (Barth and Di, 1990). Since auditory information is assumingly processed in all laminae of the auditory cortex this broader measure was sufficient for our analyses. Additionally, since the global interaction between both brain areas was the scope of the present study, further analyses were conducted on the LFPs and ECoGs averaged across all channels of the same region (cortex and striatum). The averaged signals across channels were found to explain approximately 99% of the variance between channels in the auditory cortex and about 90% of the variance of striatal electrodes. These signals were further band pass filtered (4–45 Hz, linear phase FIR filter, order = 2001).

The *coherency C*_*ij*_ (Equation 1) between two time series *x*_*i*_ and *x*_*j*_ is their complex-valued normalized cross-spectrum *S*_*ij*_ (Equation 2).

(1) $$\begin{array}{r} C_{ij}=\frac{S_{ij}\left(f\right)}{{\left(S_{ii}\left(f\right)S_{jj}\left(f\right)\right)}^{1∕2}} \end{array}$$

(2) $$\begin{array}{r} S_{ij}=\langle x_{i}\left(f\right)x_{j}{\left(f\right)}^{*}\rangle \end{array}$$

where ^*^ means complex conjugation and 〈〉 denotes the expectation value. *S*_*ii*_ and *S*_*jj*_ represent the auto spectra of the respective time-series *x*_*i*_ and *x*_*j*_.

**Figure 1:**
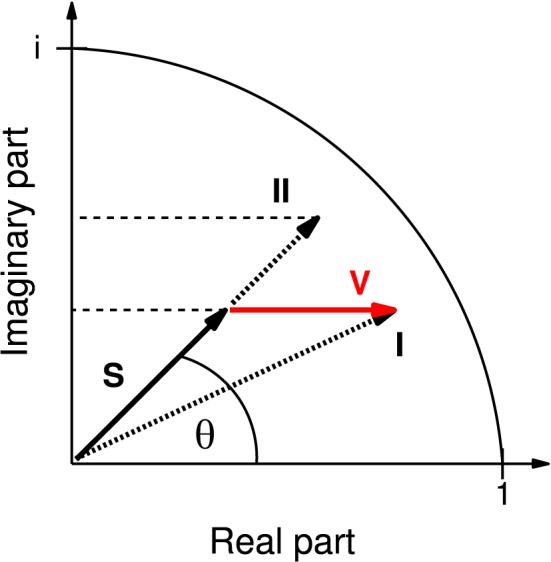
**Representation of coherency as phasor in the complex plane**. Instead of the complex-valued coherency **S**, often only its magnitude is considered (represented by the length of **S**, then called “coherence” and here denoted “mCoh”). Note that volume conduction alone, adding a real vector component V (red), can already cause an increase in mCoh. In this case (case **I**), the mCoh increase would be concomitant with a decrease of the phase angle θ of **S**. By separate analysis of the real and imaginary part of the coherency it is possible to identify mCoh increases based on concomitant increases of the real and imaginary part (case **II**). In the present study only coherence increases matching case **II** were accepted as indicating an increase in neuronal coherence free of volume conduction.

A commonly studied measure is the coherence which is defined as the absolute value (here referred to as mCoh) of the coherency (see Nolte et al., 2004). The coherency can be regarded as a phasor in a complex plane (Figure 1, S).

(3) $$\begin{array}{r} mCoh=|C_{ij}| \end{array}$$

(4) $$\begin{array}{r} iCoh=imag\left(C_{ij}\right) \end{array}$$

where *imag*(*C*_*ij*_) represents the imaginary part of the complex coherency *C*_*ij*_.

Analyzing neuronal field-field coherence of potentials recorded from different locations in the brain comes with the problem of potential influences from electrical volume conduction by third sources. This physical property affects all conducting matter, including neuronal axons. However, signals transmitted by volume conduction mathematically affect only the real part of the coherency function, not its imaginary part (*iCoh*, Equation 4). This circumstance allows for the separation of actual neuronal coherence from coherence caused by volume conduction (Nolte et al., 2004; Ewald et al., 2012).

Without volume conduction the change of mCoh is sufficient to describe changes in coherency (Figure 1). However, in a system biased by volume conduction, a third source can cause larger mCoh values due to its elevated signal strength (Figure 1, I). As volume conduction only influences the real part of the coherency, in this case the phase of the vector (Figure 1, θ) would be reduced. The increase of mCoh does therefore not necessarily mirror true increases in functional coupling.

In order to obtain a criterion for a neural coherence increase, the phase should remain constant or increase. In consequence of a constant phase and increased mCoh the iCoh increases (Figure 1, II). Hence in this study a functional coupling increase was only accepted if both coherency components, mCoh and iCoh, increased.

Coherency was calculated using Welch's method for averaging overlapping segments with a frequency resolution of the coherency spectra of 4 Hz. In order to obtain the time course of the spectra the coherency was calculated in a 500 ms long window, which was slid in step sizes of 100 ms along the time series.

#### 2.6.1. Baseline cortico-striatal coupling

Firstly a baseline measure of functional coupling without acoustical stimulation was obtained. These coherency values were compared to two simulated independent 1/f noise time series with similar spectral properties. In theory, two independent, normalized, stationary processes result in null coherency. In practice, however, some coherency remains due to finite sample sizes. This effect was used to construct the surrogate data. In a bootstrap procedure coherency values of these simulated time series were calculated 1000 times, using the same parameters as for the estimation of baseline coherency from pre-session data. The 95 percentile boundary of the bootstrapped distribution from the surrogate data was used as significance level for the real baseline coherence.

#### 2.6.2. Coherence during auditory stimulation

A second comparison was aimed at analyzing differences of coherency during acoustical presentations and baseline coherency. Only the first 3 s of each trial were used for the coherency analysis, since reaction times for Go responses were usually larger. Therefore, the first 3 s are free of artifacts and shuttling behavior. Trials with obvious movement artifacts or motor responses within this time window were rejected.

The coherency values were determined at the onsets of the first six tones (3 s after trial start) and compared to a bootstrap distribution of baseline coherency values drawn from the pre-session periods. Sample sizes were matched to trial periods (360 draws: 30 CS+ and 30 CS− presentations, each by six tones; 1000 bootstrap repetitions). Values outside the 95 percentile of the estimated distributions were declared significantly different from baseline.

#### 2.6.3. Training influences on cortico-striatal coupling

For the comparison between naïve and trained state, the coherency values during the first six CS− and CS+ presentations in every trial were averaged for the entire session. This resulted in one mCoh and one iCoh value for every session per animal, separated for the CS+ and the CS− conditions. In parametric statistical tests coherence values (mCoh and iCoh) were Fisher z-transformed.

#### 2.6.4. Modulation index

Since the period of the tone sequence was 0.5 s, mCoh and iCoh values were expected to be modulated by a frequency of 2 Hz. To obtain a modulation index, for every frequency band the envelope of the coherence was determined, offset-corrected and Fourier-transformed. The index was defined as the spectral energy contained in the 2 Hz modulation rate. The measure was normalized with the sum of spectral energies of all frequencies (range: 0–44 Hz). Consequently, the modulation index ranged from 0 (no spectral energy at 2 Hz) to 1 (entire spectral energy contained in the 2 Hz envelope frequency). The pre-session modulation index was estimated with a bootstrap procedure. Here the upper significance threshold of the modulation index was set to the 95 percentile of the bootstrapped distribution.

### 2.7. Spike-field coherence

#### 2.7.1. Spike detection and spike sorting

To relate our analyzed field-field coherence to neuronal processes, spikes in the ventral striatum were analyzed in two animals undergoing auditory discrimination training. Spike detection and sorting was conducted off-line on band-pass-filtered (300 Hz–5 kHz) continuous raw signals recorded with a sampling frequency of 25 kHz. For spike detection a magnitude threshold was estimated as described by Quiroga et al. (2004):

(5) $$\begin{array}{r} threshold=4\;*\;median\left(|x|∕0.675\right) \end{array}$$

where *x* notes raw signal during the pre-session periods of each training session. The idea behind this threshold calculation is to use the standard deviation of the signal's noise without spikes as a threshold for spike detection. Because the distribution parameters for noise without spikes are not known (particularly for high firing rates), an estimator for this standard deviation can be derived from a wavelet based denoising algorithm. This estimator depends on the median of the signal, with spikes, and a fixed factor of 0.675. The threshold was fixed for the entire session. For every threshold crossing during trials an additional window discriminator was applied. Magnitudes of the minimum and maximum peaks of identified wave shapes (cf. inlet **Figure 8**; 0.4 ms before to 1 ms after threshold crossing) defined a two-dimensional feature space. Within this feature space a semi-automatic k-means (scikits kmeans++) clustering algorithm was performed. Only defined clusters contained within this feature space were accepted as single units.

#### 2.7.2. Spike-field coherence

The spike-field coherence (SFC) was calculated according to an algorithm by Fries et al. (2001). In short, we obtained spike-triggered ECoG segments for a short time window (±250 ms) around identified spikes. Averaging these ECoG segments yielded the spike-triggered average. This average was then used to calculate the SFC, which is independent of the neuronal firing rate and the LFP power spectrum. An SFC value of 1 within a distinct frequency range indicates that all identified units spiked within the same temporal phase-relationship toward the analyzed frequency component. Null SFC for any given frequency indicates that spikes did not have any systematic phase relation to the frequency component of the ECoG.

For the construction of a significance criterion, cortical ECoG segments were drawn randomly from pre-session periods. Spike time points related to these drawn ECoG segments were chosen regardless of actual striatal spiking and matched the numbers of segments during acoustical stimulations. These random pre-session ECoG segments were then used to generate pseudo spike-field coherences (bootstrap repetition 100 times). The 95 percentile of this distribution served as threshold for non- random phase relations ships (SFC) between spike timing and ECoG.

#### 2.7.3. Software

Data analysis was done with SciPy (Jones et al., 2001), coherence calculation with Matplotlib.mlab module (Hunter, 2007), single unit clustering with scikits kmeans++ and ANOVA tests were conducted using SPSS (PASW Statistics 18, SPSS, Inc., Chicago).

## 3. Results

Mongolian gerbils (*n* = 7) underwent daily trainings in a cortex-dependent (Ohl et al., 1999) auditory Go/NoGo discrimination task. To assess cortico-striatal functional coupling during the learning process, epidural electric potentials from the auditory cortex (AC) and intracranial local field potentials from the ventral striatum were recorded during the training and analyzed using neuronal coherence measures (Supplementary Figure 2).

### 3.1. Auditory discrimination learning

**Figure 2:**
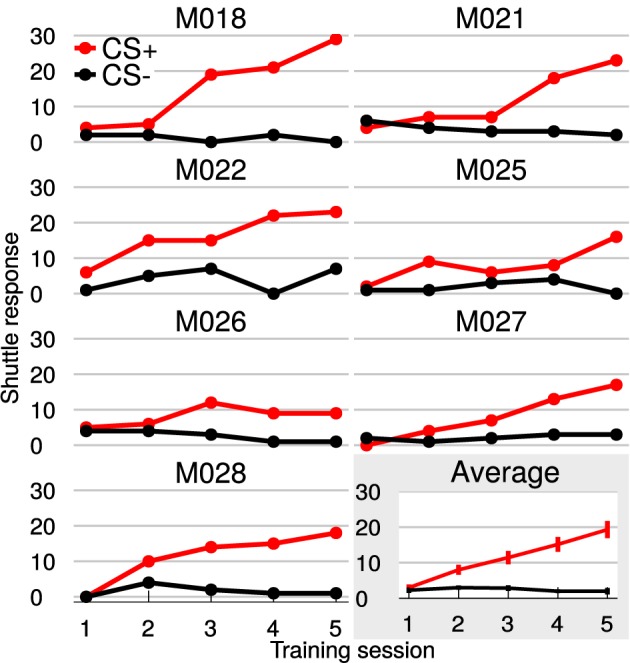
**Gerbils learned to discriminate FM-direction within 5 training sessions**. Shown are the individual learning curves with hit (red curves) and false alarm-responses (black curves). The last plot (gray background) displays the average over all animals with SEM. Animals could attain 30 hits/false alarms per session.

All animal subjects learned to discriminate rising from falling frequency modulated (FM) tones within five training sessions (Figure 2). While hurdle crossings during Go trials (CS+: hit) increased on average toward the maximum number of 30 per session (mean ± SEM in session 5: 19 ± 2), false alarm crossings during NoGo trials (CS−) stayed constant around 2 per session. Consequently, the difference between hits and false alarms, as a measure of discrimination performance, increased with training (average difference hits—false alarms, mean ± SEM in session 1: 1 ± 1; mean ± SEM in session 5: 17 ± 6).

### 3.2. Anatomical connections between the auditory cortex and ventral striatum

**Figure 3:**
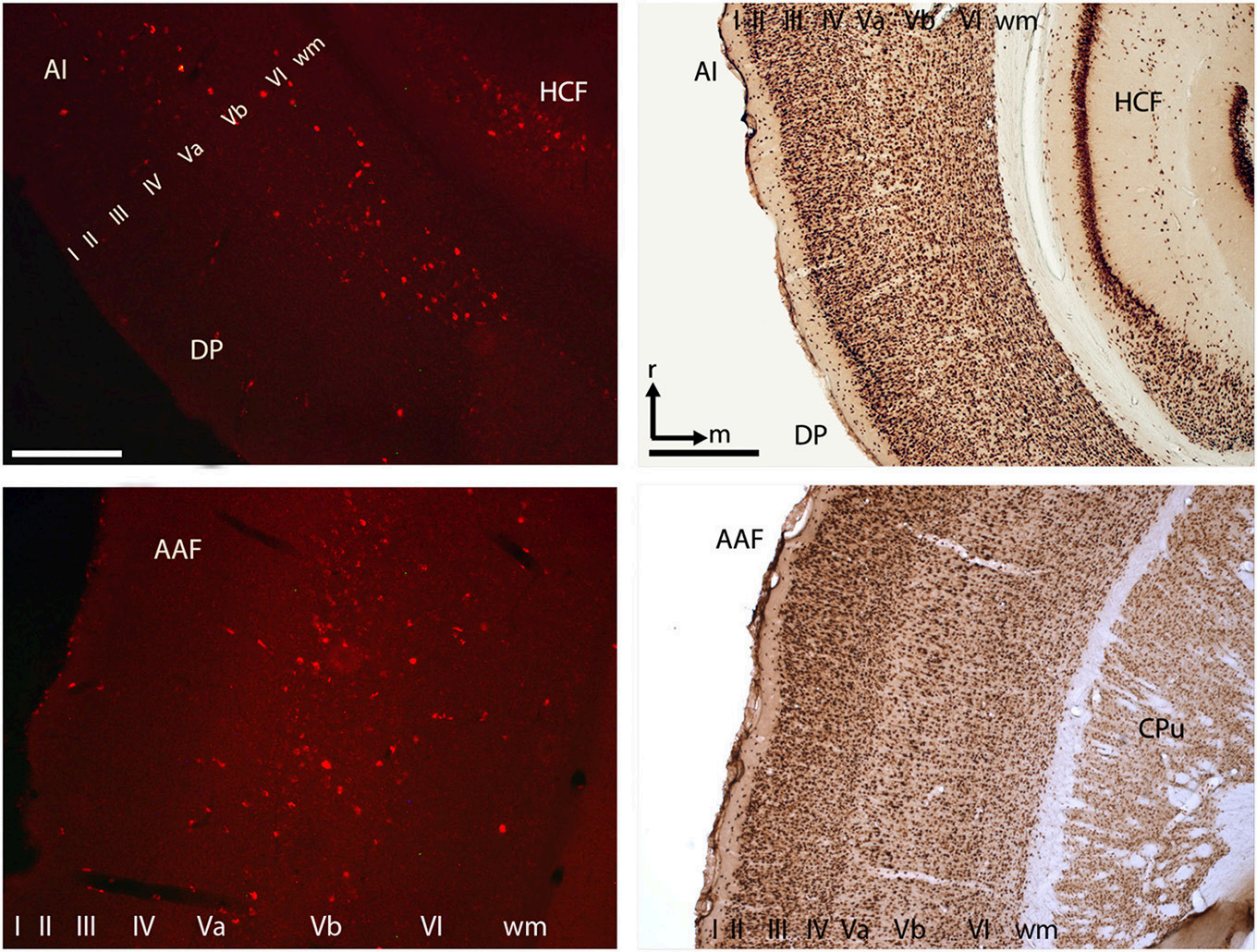
**Cortico-striatal projections originated in layer V/VI of the primary auditory cortex and within the anterior and posterior auditory fields**. Exemplary histological slices of retrogradely transported nanobeads (left) and NeuN staining (right). Scale bars left column 250 μm, right column 500 μm. AI, primary auditory cortex; DP, dorso-posterior field; AAF, anterior auditory field; CPu, caudate putamen; HCF, hippocampal formation; roman numbers I–IV, cortical layers; wm, white matter; r, rostral; m, medial.

Retrograde transport of fluorescent nanobeads demonstrated the existence of direct connections from various auditory cortical fields to the ventral striatum. Most labeled cells were seen in cortical layer 5 of the anterior auditory field, and in layers 5 and 6 of the posterior auditory fields. Projections from the primary auditory cortex were less numerous and originated mainly from layer 5 (Figure 3). With dimensions of 1.5 × 1.5 mm the utilized ECoG array covered the entire tonotopic gradient of primary auditory cortex field A1 and a smaller portion of the anterior auditory field (cf. Ohl et al., 2000).

### 3.3. Baseline coupling between auditory cortex and ventral striatum

**Figure 4:**
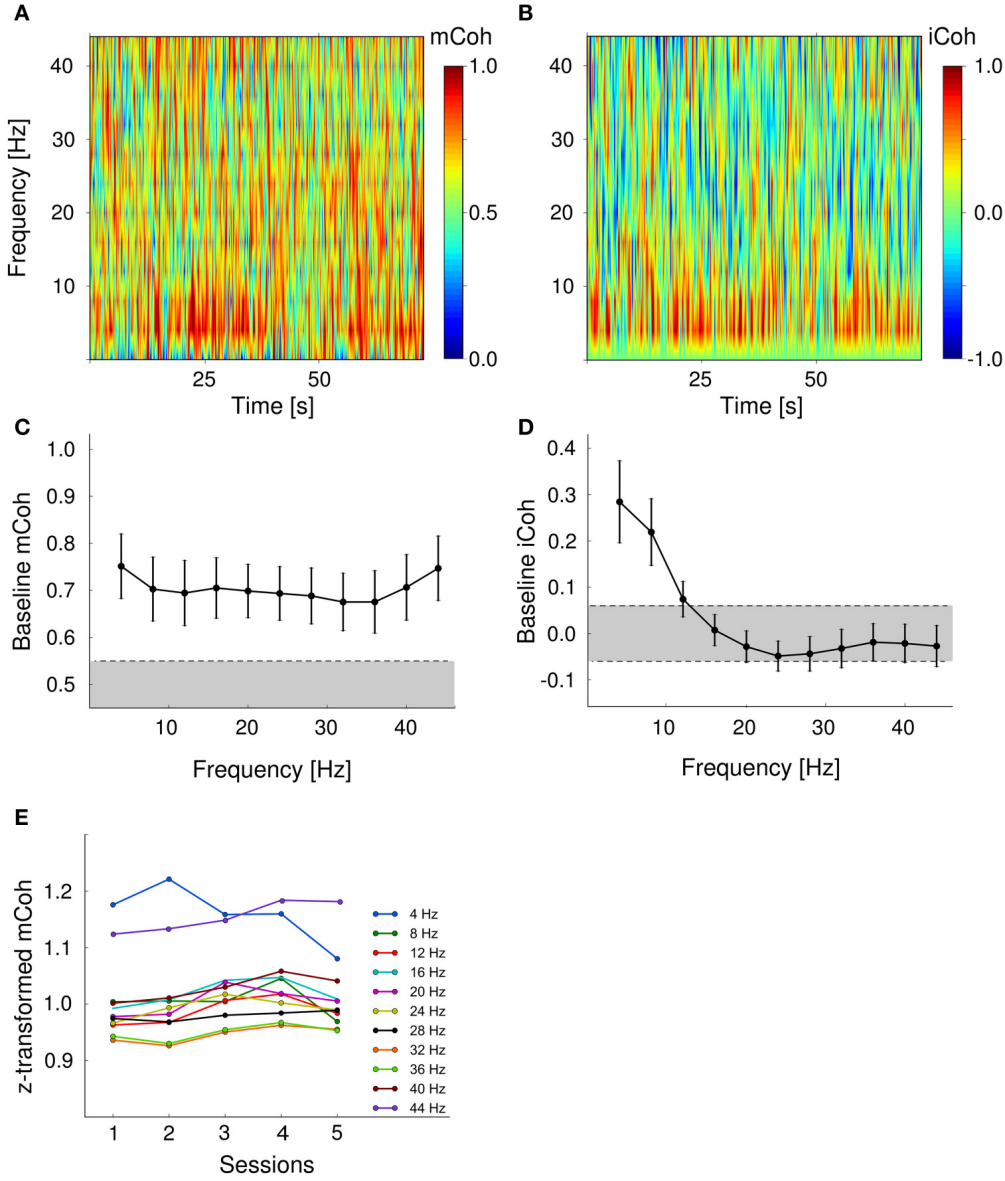
**Baseline cortico-striatal coupling remained stable over the course of training**. Baseline magnitude and imaginary coherence differed in their frequency-dependence. **(A, B)** Exemplary spectra of both coherency measures during pre-session period in one animal. **(C)** Baseline magnitude coherence was independent from frequency range. Shown are grand averages over sessions and animals. Error bars are SEMs. Gray shaded area marks values within the 95 percentile of the bootstrapped mCoh values. **(D)** Pre-session imaginary coherence was significantly increased in the low frequency range (4–10 Hz). Gray shaded area represents values within the two tailed 97.5 percentile boundaries of a bootstrapped iCoh distribution. **(E)** Grand average coherency was constant over training sessions for all frequencies analyzed.

To obtain a baseline coherency measure, pre-session periods of 75 s without acoustical stimulation prior to auditory discrimination sessions were analyzed. To ensure that found effects were not merely due to volume conduction, coherence was further delineated into magnitude coherence (mCoh) and imaginary coherence (iCoh; cf. Methods and Materials: “Field-field coherence”; Figure 1). Exemplary pre-session mCoh and iCoh spectra from one animal are displayed in Figures 4A,B. Baseline functional coupling was found to have different frequency dependencies, when mCoh and iCoh were analyzed separately for all sessions and animals (Figures 4C,D). On average, pre-session mCoh values were almost constant for frequencies from 4 to 45 Hz (Figure 4C). In addition, mCoh passed the significance criterion derived from bootstrapping mCoh values of two simulated 1/f processes in all analyzed frequency bands. This finding indicated that processes in the auditory cortex and the ventral striatum were not independent, even without acoustical stimulation.

The imaginary coherence, that reflects the phase relationship between two time series, only exceeded the significance threshold in the lower frequency range (4–12 Hz; Figure 4D). iCoh values in higher frequency bands were within the 97.5% range of the bootstrapped distribution and therefore indicated an in-phase relationship between signals from the AC and striatum. An increased mCoh in combination with constant iCoh represents a typical pattern that is potentially produced by volume conductance (cf. Methods and materials: “Field-field coherence”; Figure 1). Therefore, during pre-session periods, cortico- striatal neural coupling in frequency bands larger than 12 Hz could not be distinguished from spurious magnitude coherence caused by volume conductance.

Finally, we tested whether baseline cortico-striatal coupling was changed during auditory discrimination training as an indicator of potential stimulus-independent effects that interfere with task-specific coupling changes. Session effects on pre-session mCoh were tested with a repeated-measure ANOVA (within subject factors: session and frequency band). There was no significant main effect of the factor “training sessions” on pre-session mCoh [*F*_(4, 24)_ = 1.1, *p* = 0.38], but the factor “frequency band” appeared to show an influence on mCoh [*F*_(10, 60)_, *p* < 0.001] during pre-session periods. *Post-hoc* paired *t*-tests revealed that this was mainly due to overall higher mCoh values in the 4 and 44 Hz band, compared to the other frequencies (Figure 4E). There was no interaction between factors “training session” and “frequency band” [*F*_(20, 240)_ = 0.951, *p* = 0.56].

### 3.4. Auditory stimulus presentation modulated cortico-striatal coherence

**Figure 5:**
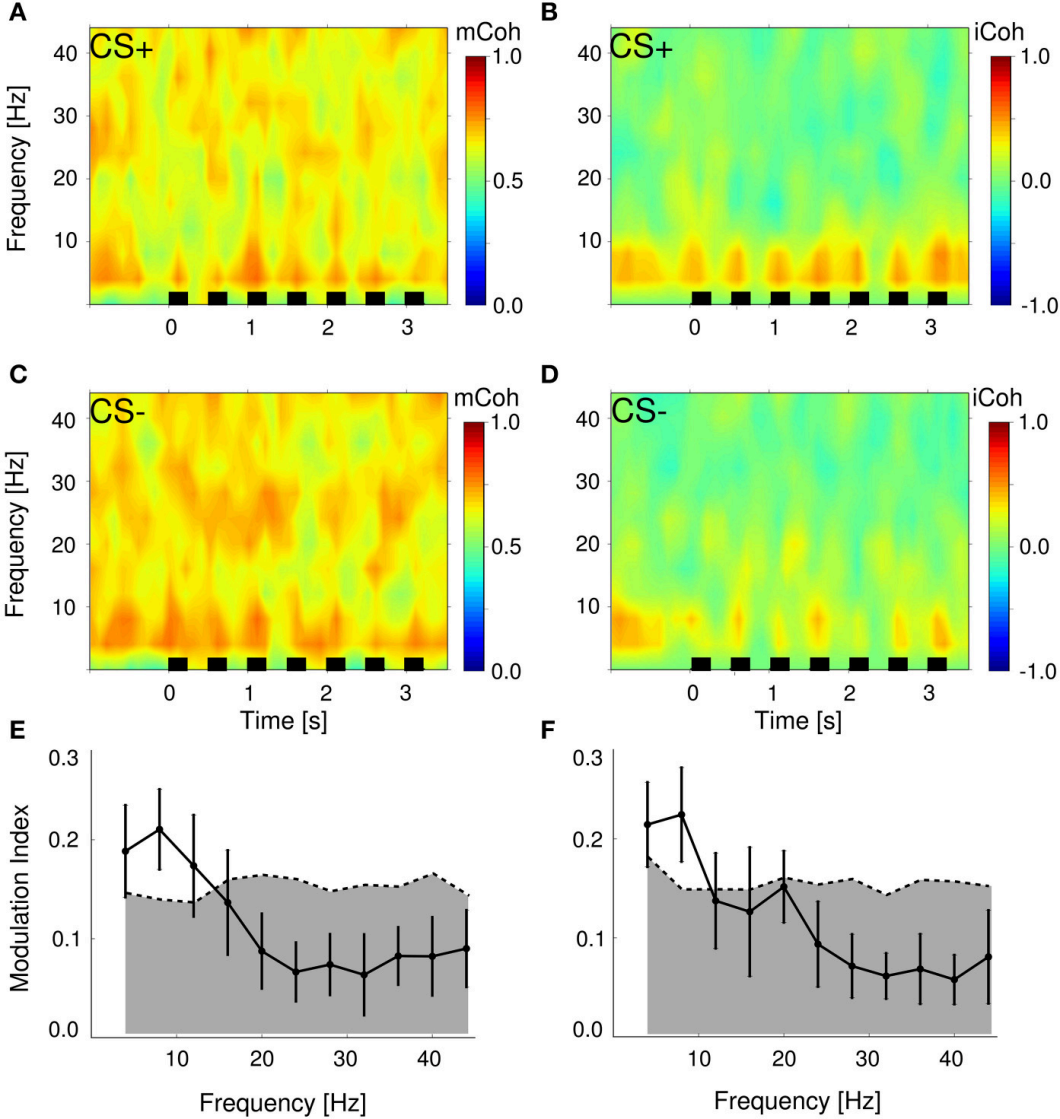
**Stimulus presentations significantly modulated magnitude and imaginary coherence during trials**. **(A,C)** Average magnitude coherence spectra of one session from the same animal as in Figure 4; trial conditions (CS+ and CS−) were pooled. Black bars represent stimulus onsets and duration. **(B,D)** Same display as in **(A,C)** for the iCoh. **(E,F)** Modulation indexes were significantly increased in the low frequency range 4–10 Hz for mCoh **(E)** and 4–8 Hz iCoh **(F)**. Depicted are grand averages with SEMs. Gray shaded areas mark 99 percentile boundary of a bootstrapped distribution of modulation indexes from pre-session periods for both coherency measures.

During auditory discrimination training each trial was started with the presentation of the conditioned stimulus, a sequence of upward or downward frequency-modulated tones (CS+: 1–2 kHz; CS−: 2–1 kHz). Spectra of mCoh and iCoh for one representative session during CS+ and CS– presentations are shown in Figures 5A–D. In this figure, both coherency measures were aligned with respect to trial onsets. Spectra were averaged over trials (30 CS+ and 30 CS−).

To analyze changes due to auditory stimulus presentations, the averaged evoked mCoh and iCoh per session were compared to the average of the mCoh values from the pre-session period. For instance in the 8 Hz frequency band in 71% of all sessions the CS− evoked mCoh was larger than the mCoh during pre-session period and in 79% of all sessions the CS+ evoked mCoh was larger than during pre-session period. However, the CS− evoked mCoh was only in 24% (*t*-test, *p* < 0.05) and the CS+ evoked mCoh was in 41% (*p* < 0.05) significantly larger than the corresponding pre-session mCoh (for the numbers in all frequency bands and the iCoh see Supplementary Figure 1 and Supplementary Tables 1, 2).

Despite the fact that in most sessions mCoh during tone presentations was not significantly larger than the pre-session mCoh, the sequence of tones induced a notable temporal structure on both coherency measures (Figures 5A–D). To obtain a quantitative measure, a modulation index was calculated, that expressed the amount of spectral energy contained within the repetition rate of the auditory stimulation (2 Hz). Figures 5E,F display this modulation index as a function of frequency bands. The modulation index of both, the mCoh and the iCoh during stimulus onsets, were significantly larger than modulation index of pre-session periods in frequency bands below 10 Hz. These results confirmed that the temporal dynamics of mCoh and iCoh values were particularly influenced in the lower frequency ranges by the periodicity of the CS tone trains.

### 3.5. Cortico-striatal functional coupling increased for CS+ presentations during auditory discrimination training

All animals showed significant improvement of discrimination performance over the course of training (Figure 2). In order to increase statistical power coherence values from sessions 1 and 2 were pooled into “early sessions” and coherence values from sessions 4 and 5 were pooled into “late sessions.” Go- and NoGo-trials (i.e., CS+ and CS− presentations) were analyzed separately. Both trial categories were tested with repeated-measures ANOVA for the effects of factors session (levels: early and late) and frequency band.

**Figure 6:**
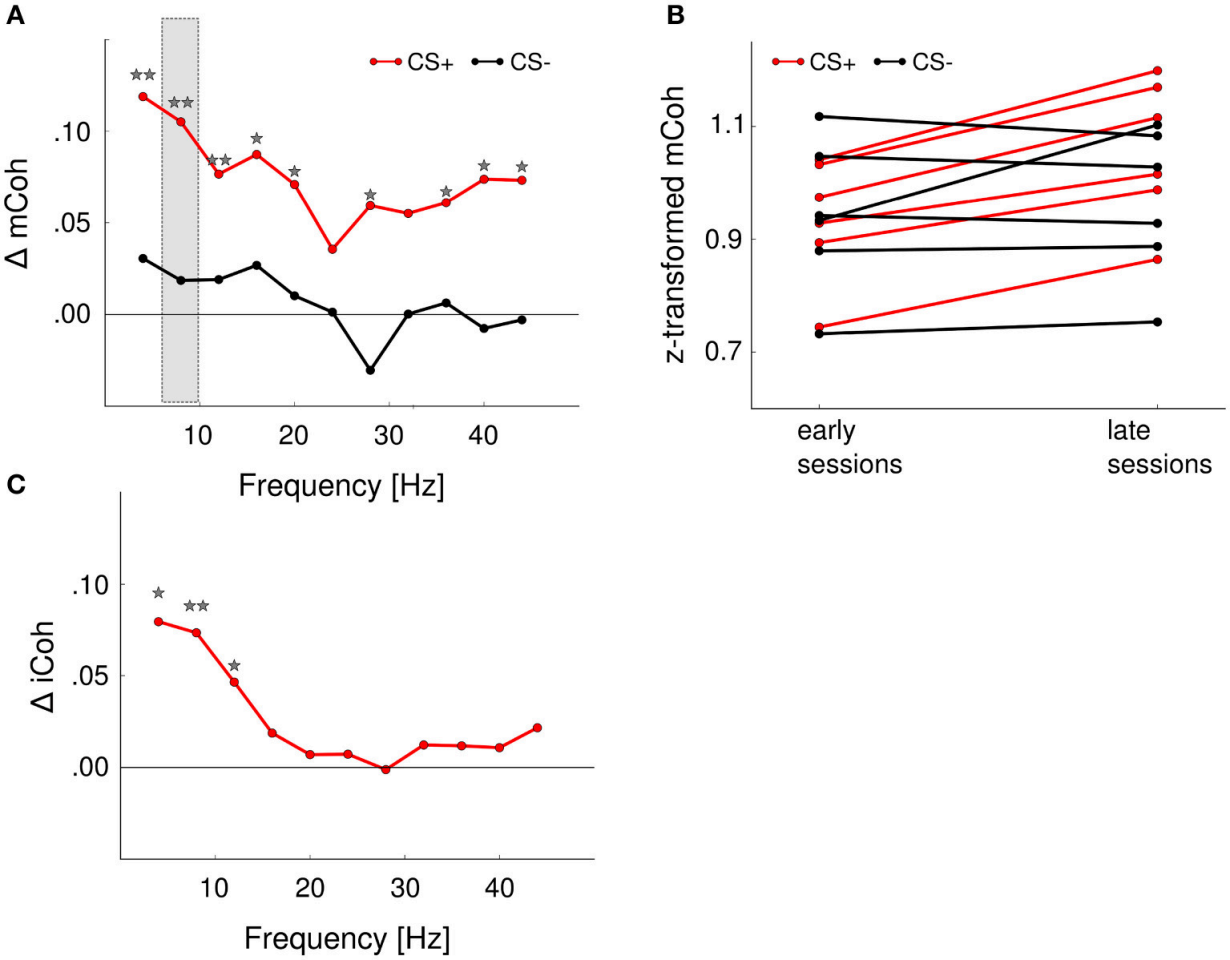
**Auditory discrimination learning specifically increased functional cortico-striatal coupling**. **(A)** mCoh increases from early to late training sessions for CS+ presentations were detected in all frequency ranges from 4 to 40 Hz. Shown are the average differences from early to late sessions of all animals. ^*^p < 0.05, ^**^p < 0.01, *post-hoc* paired *t*-tests. Shaded rectangle shows values displayed in **(B)**. **(B)** Onset mCoh during CS+ trials increased from early to late training sessions (red lines), while presentations of CS– tones did not alter onset mCoh values during training (black lines). Shown are the average z-transformed mCoh values of the 8 Hz frequency band in early and late sessions of individual animals (*n* = 6). **(C)** iCoh values significantly increased in the 4–10 Hz frequency range for CS+ presentations. CS− iCoh values were not further analyzed, as mCoh values were not changed during training.

The mCoh values during CS+ presentations increased significantly over sessions [Figure 6A; repeated-measures ANOVA; frequency band: *F*_(10, 60)_ = 12.3, *p* < 0.001; session: *F*_(1, 6)_ = 35, *p* = 0.001; frequency band × session *F*_(10, 60)_ = 0.94, *p* = 0.5]. A *post-hoc t*-test [−6.4 < *t*_(6)_ < −1.3, 0.00067 < *p* < 0.234 for CS+, for CS− all *p* > 0.05] revealed that the onset mCoh increased significantly in several frequency bands from early to late sessions (Figures 6A,B). To meet the criterion for neuronal coherence increase free of contributions from mere volume conduction, the change of the iCoh during CS+ presentations over sessions was tested with identical factors. There was a trend-wise main effect of factor session on iCoh, but a significant frequency band × session interaction effect [repeated-measures ANOVA; frequency band: *F*_(10, 60)_ = 120.5, *p* < 0.001; session: *F*_(1, 6)_ = 4.89, *p* = 0.07; frequency band × session: *F*_(10, 60)_ = 3, *p* = 0.004]. A *post-hoc t*-test revealed that for the iCoh values, significant session-related changes were restricted to low frequency bands [frequency band 4 Hz, *t*_(6)_ = −3.17, *p* = 0.02; 8 Hz, *t*_(6)_ = −3.76, *p* = 0.009; 12 Hz, *t*_(6)_ = −2.4, *p* = 0.0499; all other frequency bands: *p* > 0.05; Figure 6C]. Combined analysis of mCoh and iCoh revealed that for higher frequencies significant changes of the mCoh were not paralleled by corresponding iCoh changes. Therefore, coherency changes in the higher frequency bands cannot be considered free of volume conduction (Figures 6A,C). The mCoh during CS− presentations showed no significant changes with sessions [Figure 6A; repeated- measures ANOVA; frequency band: *F*_(10, 60)_ = 7.1, *p* < 0.001; session: *F*_(1, 6)_ = 0.25, *p* = 0.64; frequency band × session: *F*_(10, 60)_ = 0.48, *p* = 0.89]. Since the criterion for coherence increases was not met, iCoh changes for CS− presentations were not tested. Altogether, these results indicated a specific increase of functional cortico-striatal coupling in frequency bands up to 12 Hz during CS+ presentations accompanied by increased discrimination performance during learning of the Go/NoGo-task.

### 3.6. Cortico-striatal coupling during shuttling behavior

**Figure 7:**
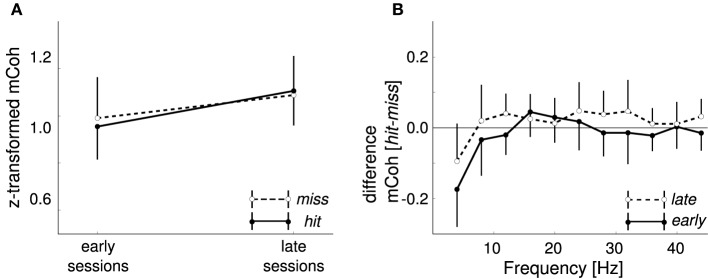
**Different motor responses did not cause the increase in CS+ onset coherencies**. **(A)** Onset magnitude coherence (mCoh) was not differentially changed during CS+ presentations when split into shuttling (hit) and staying (miss) responses. Shown are grand averages for late and early sessions of miss trials (white dots) and hit trials (black dots) in the 8 Hz frequency band. **(B)** Differences between hit and miss trials from early and late training sessions were not frequency dependent. Values ranged around zero for all frequencies except 4 Hz; here the difference was negative, indicating higher mCoh values for non-jump responses. Error bars represent SEM.

Along with the training the number of hits increased while the number of misses decreased, whereas the number of false alarms remained approximately constant (Figure 2). A potential explanation for the increase of coherence during CS+ trials over sessions is that it simply reflects motor or premotor activity. In this case an increase of the averaged mCoh with training sessions would result from more frequent shuttling behavior. To exclude this possibility, mCoh values were compared for hit and miss trials during the CS+ trial condition. A repeated-measure ANOVA (factors: “frequency band,” “sessions” with levels early and late sessions, “response” with levels hit and miss) revealed that there was no statistically significant difference of mCoh values for the factor response [*F*_(1, 6)_ = 0.17, *p* = 0.69]. A *post-hoc* paired *t*-test between hit and miss trials in every frequency band showed no significant differences between their mCoh values during early and late sessions (*p* > 0.05; Figure 7). The difference between mCoh values during hit and miss conditions in the 4 Hz frequency band proved to be statistically significant during earlier sessions, however [*t*_(6)_ = −*4.1, p* = 0.007]. Notably, here the mCoh during the miss condition surmounted the mCoh in the hit condition. Taken together, these results support that the overall increased mCoh during the presentation of CS+ tones in later sessions cannot be explained by a motor-related confound increasing with the number of hits.

### 3.7. Auditory stimulation also modulated cortico-striatal spike-field coherence

Spike-field coherence (SFC) is often applied as an indicator that field-field coherence is not merely a result of volume conductance. In this respect we utilized the SFC here, additionally to the increase of the iCoh, which is in our view the more conservative measure. As it is experimentally difficult to record from identical single neurons over several sessions or days, we related neither the SFC nor the peristimuls time-histograms to the learning process, but estimated the SFC only in one session at the later learning stages for two animals.

**Figure 8:**
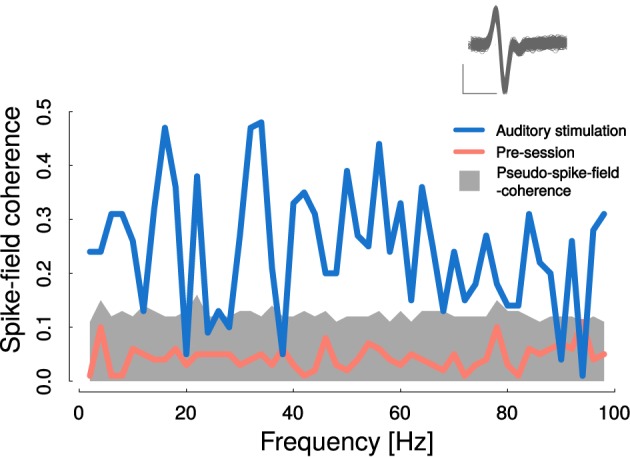
**Cortico-striatal spike-field coherence was elevated during auditory stimulation**. Auditory stimulation led to significantly increased spike-field coherence in nearly all frequency ranges from 1 to 100 Hz (blue line). Gray area represents 99 percentile-bound distribution of bootstrapped pseudo spike- field coherences from pre-session recordings. Average pre-session spike-field coherence is represented by the magenta curve. Inlet shows recorded spike shapes; scale: x: 0.5 ms, y: 0.15 mV.

Figure 8 displays SFC during auditory stimulation and during pre-sessions. Pre-session SFC (magenta curve) ranged below the significance threshold derived from pseudo spike times (see Materials and methods) and was smaller than SFC during auditory stimulations (CS+ and CS− trials pooled). During auditory stimulation SFC clearly surmounted the estimated threshold in the entire frequency range from 2 to 100 Hz, similar to the baseline mCoh (Figure 4C). However, SFC can also be influenced by volume conduction. Based on the change of the iCoh (Figure 4D) we therefore concluded, that theta band-SFC resembled functional coupling, whereas influences of volume conductance could not be ruled out in higher frequency bands.

## 4. Discussion

In this study we demonstrated that learning of an auditory-cued discriminative Go/NoGo avoidance response is associated with a strengthening of functional coupling between the auditory cortex and ventral striatum. Hence, even stimuli evoking largely overlapping tonotopic representations in auditory cortex can lead to markedly different behaviors in the course of discrimination learning. These differential changes were also reflected in the different changes of cortico-striatal coupling.

### 4.1. Baseline and stimulus induced functional coupling between auditory cortex and ventral striatum

We observed a significant coherence between auditory cortex and ventral striatum already during time periods without acoustical stimulation and a stimulus induced high correlation between the temporal modulation of the coherence spectra and the tone sequence. This finding indicates that both brain areas are functionally coupled. Furthermore, we could qualitatively show direct anatomical connections from the auditory cortex to the ventral striatum (Figure 3). Various monosynaptic efferent connections from auditory cortex to the ventral striatum have also been demonstrated by our group for gerbils (Budinger et al., 2000, 2008) and in other species such as monkeys and cats (Yeterian and Pandya, 1998; Winer, 2005). In addition, the non-random field-field coherence results were qualitatively matched by significant non-random spike-field coherence between ventral striatum and auditory cortex during auditory stimulation (Figure 8). While monosynaptic cortico-striatal coupling represents a parsimonious rationale behind the reported changes in coherence between auditory cortex and ventral striatum, indirect polysynaptic connections or a common neuronal input could account for our findings, as well. Candidates for this common source are thalamic nuclei and midbrain structures (McHaffie et al., 2005; Haber and Calzavara, 2009; Smith et al., 2011). Adding to this, Schulz et al. (2009) have shown that short-latency visual input can reach the striatum not only via the tecto-nigro-striatal route but also through direct tecto-thalamo-striatal projections.

### 4.2. Selective increase of cortico-striatal coupling with auditory discrimination learning

During the discrimination task Mongolian gerbils learned to shuttle in response to a Go stimulus (a rising frequency-modulated tone) and remain in the current compartment of the shuttlebox during NoGo-stimulus presentations (falling frequency-modulated tones). All animals had reached significant discrimination performance after 5 sessions.

During the course of learning, functional coupling, measured as cortico-striatal field-field coherence, selectively increased during the presentation of CS+ stimulus (Go) but not during the CS− tones (NoGo). The increased functional coupling cannot be merely explained by motor responses (shuttling behavior) that also increased during the course of training due to increased hit rate. These findings are in support of the theory that learning differential behavioral responses to conditioned stimuli requires the formation of neuronal populations that are then differentially recruited during the task accordingly (Schultz, 2001; Frank et al., 2004; Amemori et al., 2011).

The striatum receives projections not only from the auditory cortex but from all other sensory cortical structures (McGeorge and Faull, 1989; Glynn and Ahmad, 2002). It is not novel that the striatum, especially its ventral partition, differentially encodes stimulus-reward associations (Goldstein et al., 2012; Atallah et al., 2014), displays responses to auditory stimuli (McGeorge and Faull, 1989; Cromwell et al., 2007; Woldeit et al., 2012; Zhao et al., 2015) and to areas involved in processing other sensory modalities (Glynn and Ahmad, 2002; Nagy et al., 2005, 2006; Schulz et al., 2009; Reig and Silberberg, 2014). To our knowledge the present study is among the first to investigate plastic changes between auditory cortex and striatum during a learning task and corresponding studies are still lacking for other sensory modalities.

The reason that the evoked coherence did not increase for CS– stimuli over training could be the differential reinforcement schedule. At the start of the behavior training stimuli have an aversive connotation, since they are followed by a footshock, in the case the animal commits an error. Yet, only during Go stimulus presentation cortico-striatal coherence was found to be increased. This stimulus selective alteration of coherence can be explained on the basis of the two-factor theory of avoidance learning (Mowrer, 1951). Accordingly, the CS+ first becomes associated with the foot shock and elicits fear in the subject. Later during learning avoidance responses appear and become associated with the termination of the CS+ resulting in a subsequent release of fear, which produces an internal reinforcement that is associated with dopaminergic signaling during successful avoidance (Ilango et al., 2010, 2012; Dombrowski et al., 2013; Ilango et al., 2014) potentially modifying cortico-striatal synaptic strengths (Sutton and Barto, 1998; Schultz, 2001). While this scenario would assumingly be similar for false alarm trials their low counts causes the coherency measure during CS− presentations to be dominated by correct rejections. As correct rejection responses had no direct consequences no internal reinforcement and consequently no change of functional cortico-striatal connectivity was expected.

Dopamine as well as other learning related neuromodulators, like norepinephrine (Shepard et al., 2015), acetylcholine (Kilgard and Merzenich, 1998; Gaucher and Edeline, 2015) and serotonin may also play a role in this plasticity, especially in the auditory cortex (for a review see Thiel, 2007). As a matter of fact cortical plastic reorganization might have been a factor contributing to our found stimulus-specific modulation of functional cortico-striatal coupling. In addition to this, dopamine and norepinephrine status have been shown to modulate cortico-striatal synchrony and coherence, especially in the theta range (Costa et al., 2006; Dzirasa et al., 2010).

We would like to emphasize that for the the suggested mechanism for selectively increased functional coupling during a discrimination task, it is crucial that the two stimuli elicit two different neural representations in the auditory cortex and therefore create two distinct subsets of cortical neurons in terms of a population coding. These two different populations of cortical neurons can be based on cortical tonotopy, if pitch serves as conditioning feature as in the Xiong et al. (2015) study, or on different functional properties of cortical cells. The two utilized auditory conditioned stimuli of the present study possessed identical spectral content but differed in their temporal structure; their identical spectral energy has been shown to cause activation of neurons in the same tonotopic areas of the auditory cortex (Budinger et al., 2000; Ohl et al., 2000; Budinger et al., 2008). On the other hand, frequency-modulation direction sensitive neurons have been identified at the single unit level in all stages of the auditory pathway (for a review see Rabang et al., 2015). However, the topographical organization of these cells is still under debate (Mendelson et al., 1993; Ohl et al., 2000; Zhang et al., 2003; Godey et al., 2005; Atencio et al., 2007; Trujillo et al., 2011). It appears most likely that the CS+ and CS− of the present study activate subpopulations of neurons that belong to different neuronal classes in terms of FM-direction selectivity, but that are almost equally distributed in the tonotopic range of the used frequency range. The reinforcement of the conditioned stimulus and the related behavior (reward or punishment) would then modulate the synaptic strength between active cortical presynaptic neurons and active striatal postsynaptic neurons (Schultz, 1998). This mechanism allows for a flexible, behavior dependent functional coupling between cortex and striatum. Xiong et al. (2015) speculated that their results were not limited to stimuli well separated by spectral properties and therefore activated only by cortical subpopulations well separated on the tonotopic map. With the use of frequency modulated tones and the hypothetical activation of direction selective neurons we showed their speculations were correct.

Validation of the coherency measures against potential influences of volume conduction showed that the latter could not be excluded in frequency bands above 12 Hz. Therefore, we can only make conclusions about the frequency range below 12 Hz, which mostly corresponds to the theta band. The theta rhythm encompasses frequencies between 4 and 12 Hz and is well known for its role in coupling long range connection between different brain structures (Fries, 2005; Kay, 2005; DeCoteau et al., 2007; Tort et al., 2008; van der Meer and Redish, 2011). Furthermore, the ventral striatum is known for prominent theta oscillations that are coherent across large parts of the striatum during cue instructed behaviors (Berke et al., 2004; DeCoteau et al., 2007; van der Meer and Redish, 2011) and locally generated theta oscillations have also been identified in the auditory cortex (Lakatos et al., 2005, 2007). *In vitro* co-cultures of cortical and striatal cells also display peak synchrony around 7–15 Hz (Plenz and Aertsen, 1996) and theta has been identified as good candidate to couple the striatum to other brain areas. Adding to this high-voltage spindles are a different physiological feature of the basal ganglia that falls into the frequency range of interest (5–13 Hz, Berke et al., 2004). These might represent critical cortical afferents to the striatal medium spiny neurons. They correspond to behavioral episodes of quiet wakefulness in which animals are responsive to tactile, auditory and visual stimuli (Dejean et al., 2007) and vanish at the onset of movement (Berke et al., 2004). Given these findings, we believe that theta oscillations represent a prominent mechanism that could orchestrate the coupling between striatum and auditory cortex.

To our knowledge there is only one previous study measuring functional coupling between AC and striatum during learning. In a study of auditory Pavlovian discrimination in cats Popescu et al. (2009) found no change of coherence in the gamma range between auditory cortex and striatum during learning. Although they found a marked peak of auditory cortico-striatal coherence in the low frequency range, they only investigated the change of functional gamma coupling during their training. In contrast to the present study their behavioral task was appetitive and reinforcement was given independent of the cat's behavior, which heavily limits the comparability of both studies. Fritz et al. (2010) trained ferrets in a comparable conditioning avoidance task and found that the coherence in the frequency band between 10 and 20 Hz between auditory and prefrontal cortex was significantly reduced if the animals were engaged in the task compared to passive listening. However, there were major differences to our study. Fritz et al. (2010) did not compare coherence of naïve and trained state of the animals but investigated how coherence changed between passive and active listening. The observed reduction of cortico-cortical coherence occurred after only 10 trials (i.e., several seconds), which is a much shorter time scale compared to our observed changes.

It remains an open question and direction for future research whether the coherence between cortex and striatum depends on task-engagement and whether the timing of modulation of coherence depends on the reinforcement schedule of the task. While a most recent study has shown increased caudate-putamen neuronal activity in trained cats during a passive listening phase (Zhao et al., 2015), task-engagement and premotor activity are hard to distinguish from perceptual processing in a learning experiment. Even if no motor activity can be observed, e.g., because the animals are in a different environment or reinforcement-context, it may be possible that the conditioned stimuli still elicit premotor activity. In that sense a trained animal is always engaged in the learned task. In future studies it would be interesting to compare our results to an appetitive paradigm, such as a lever-press or T-Maze task. Our experiments could also be extended by switching the contingencies of the CS-stimuli (Popescu et al., 2009); if one would not find comparable increases in cortico-striatal functional coupling during such a reversal, the differential nature of operant and Pavlovian learning would become clearer.

### 4.3. Conclusions

Here we demonstrate that auditory cortex and ventral striatum interact throughout an auditory learning task, specifically that their coherence is strengthened throughout behavioral training. We have shown that even stimuli activating largely overlapping areas in sensory cortex can give rise to different patterns of cortico-striatal coherence when these stimuli are associated with different behaviors. Cortical neuronal subpopulations activated by spectrally identical stimuli assumingly project to spatially overlapping target regions in the striatum, as well. Hence our results show that learning modifies functional coupling, allowing stimulus specific and behavior specific change of functional coupling, even if the relevant cortico-striatal connections overlap spatially. This mechanism is flexible and allows mapping of stimuli with complex cortical neuronal representations to distinct behaviors.

## Author contributions

AS, MW, and FO designed the study, evaluated the results and wrote the manuscript. AS, MW, AG, and KS performed the experiments. AS, MW, KS, and AG analyzed the data.

## Funding

This work was supported by grants from the Deutsche Forschungsgemeinschaft (DFG) and the German Federal Ministry of Education and Research (BMBF).

### Conflict of interest statement

The authors declare that the research was conducted in the absence of any commercial or financial relationships that could be construed as a potential conflict of interest.

## Abbreviations

ANOVA: analysis of variance
CS+/−: conditioned Go/NoGo stimulus
ECoG: electrocorticogram
iCoh: imaginary coherence
LFP: local field potential
mCoh: magnitude coherence
PBS: phosphate-buffer
SEM: standard error of the mean
SFC: spike-field coherence.
