# Supplementary material for: Selective Increase of Auditory Cortico-Striatal Coherence during Auditory-Cued Go/NoGo Discrimination Learning
Source: Front Behav Neurosci. 2016 Jan 11;9:368. doi: 10.3389/fnbeh.2015.00368 (PMC4707278; doi:10.3389/fnbeh.2015.00368)
Supplement: Supplementary file 1 [file Presentation1.pdf]

Supplementary Material: Selective increase of auditory cortico-striatal coherence during auditory-cued Go/NoGo discrimination learning

Andreas L. Schulz [1,*], Marie L. Woldeit [1], Ana I. Gonçalves [1,2], Katja Saldeitis [1] and Frank W. Ohl [1,2,3]

*Correspondence:
Dr. Andreas L. Schulz
andreas.schulz@lin-magdeburg.de

1 SUPPLEMENTARY TABLES AND FIGURES

Supplementary Table 1: Number of sessions in which CS-evoked magnitude of coherency (mCoh) was larger or significantly larger (t-test, $p < 0.05$) than pre-session cortico-striatal coherence. The total number of sessions are 34 (7 animals x 5 session; session 5 of one animal was excluded due to poor signal quality).

Frequency band [Hz]	CS-	CS+	CS- ($p < 0.05$)	CS+ ($p < 0.05$)
4	20	16	6	8
8	24	27	8	14
12	20	22	6	14
16	22	20	4	5
20	18	15	5	4
24	18	21	0	3
28	25	23	2	5
32	24	20	1	4
36	22	20	4	1
40	20	22	2	1
44	17	20	4	1
48	18	18	2	2

Supplementary Table 2: Number of sessions in which CS-evoked imaginary part of coherency (iCoh) was larger or significantly larger (t-test, $p < 0.05$) than pre-session cortico-striatal coherence. The total number of sessions are 34 (7 animals x 5 session; session 5 of one animal was excluded due to poor signal quality).

Frequency band [Hz]	CS-	CS+	CS- ($p < 0.05$)	CS+ ($p < 0.05$)
4	17	15	13	14
8	23	29	9	13
12	25	32	2	12
16	26	22	4	3
20	20	18	0	1
24	19	16	1	2
28	23	17	1	1
32	23	21	2	2
36	21	20	2	0
40	26	26	4	6
44	23	21	4	2
48	22	22	3	1

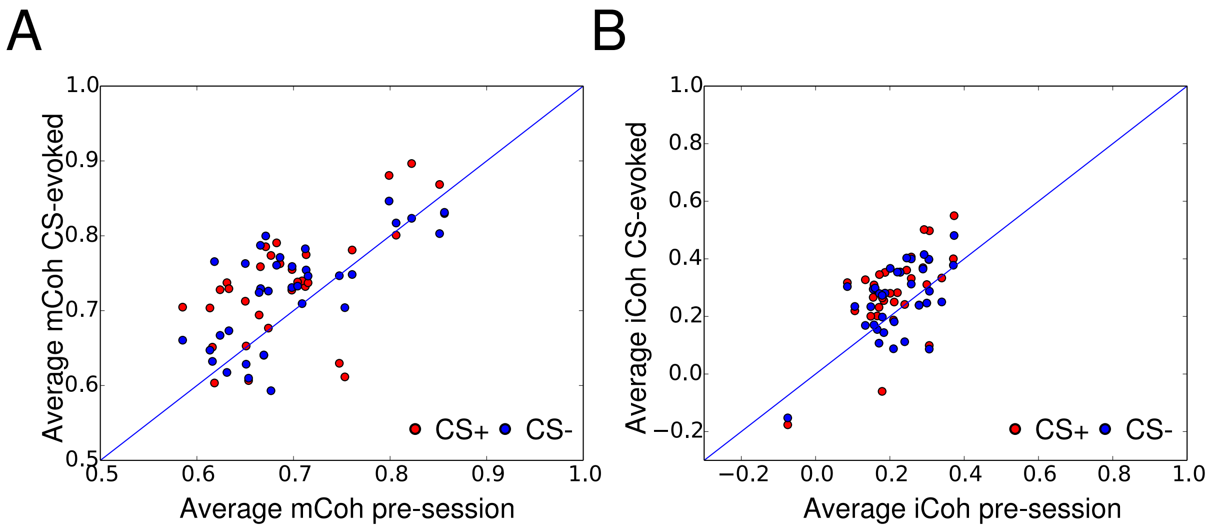

Supplementary Figure 1: Session average of evoked coherency versus pre-session coherency. **(A)** Magnitude coherency (mCoh), **(B)** imaginary part of coherency (iCoh)

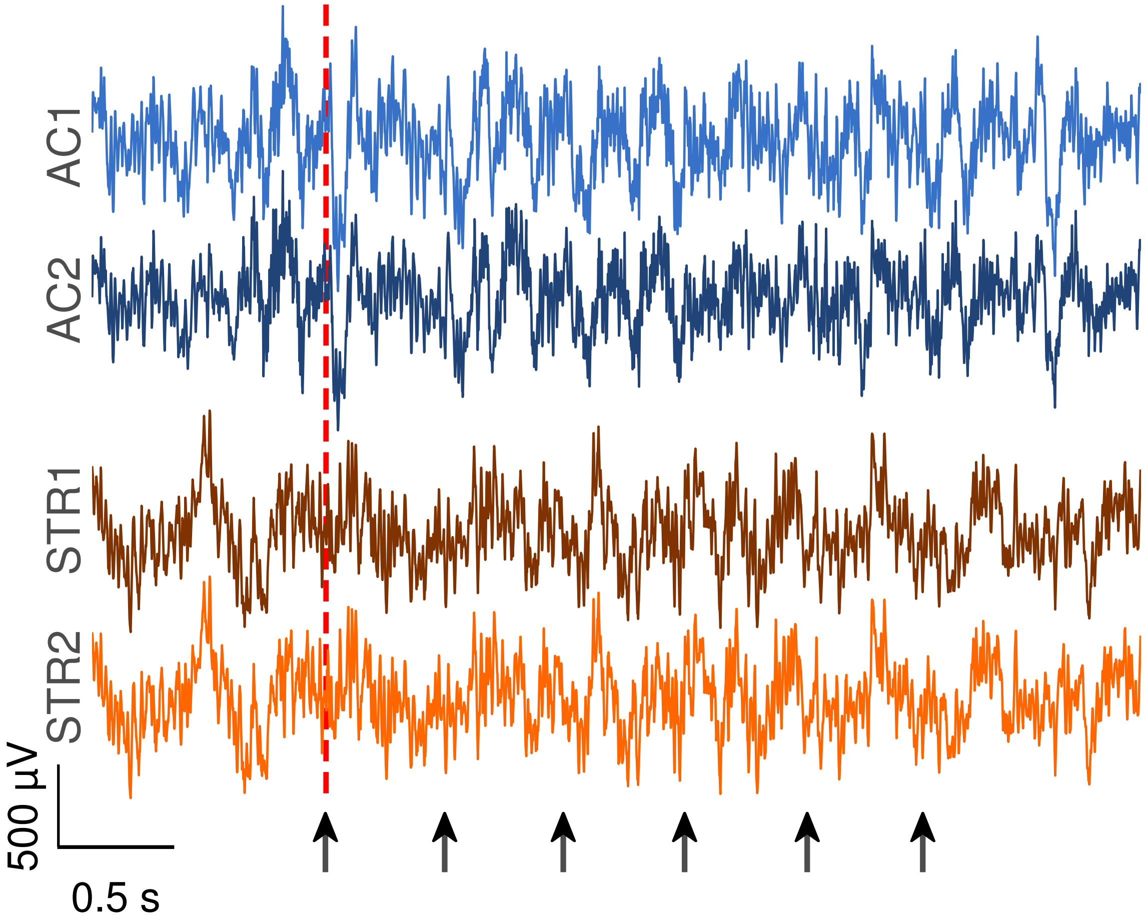

Supplementary Figure 2: Exemplary raw LFP traces of two auditory cortex and two striatal channels during training. Red dashed line: trial onset; arrows CS presentations.
